# Comparison of primary care experiences among adults in general outpatient clinics and private general practice clinics in Hong Kong

**DOI:** 10.1186/1471-2458-10-397

**Published:** 2010-07-06

**Authors:** Samuel YS Wong, Kenny Kung, Sian M Griffiths, Tanya Carthy, Martin CS Wong, Su V Lo, Vincent CH Chung, William B Goggins, Barbara Starfield

**Affiliations:** 1 School of Public Health and Primary Care, The Chinese University of Hong Kong, Hong Kong SAR, PR China; 2 Strategy and Planning, Hospital Authority Headquarter, Hong Kong SAR, PR China; 3 School of Public Health, Johns Hopkins University, Baltimore, USA

## Abstract

**Background:**

The main goal of Hong Kong's publicly-funded general outpatient clinics (GOPCs) is to provide primary medical services for the financially vulnerable. The objective of the current study was to compare the primary care experiences of GOPC users and the users of care provided by private general practitioners (GPs) in Hong Kong via a territory-wide telephone survey.

**Methods:**

One thousand adults in Hong Kong aged 18 and above were interviewed by a telephone survey. The modified Chinese translated Primary Care Assessment Tool was used to collect data on respondents' primary care experience.

**Results:**

Our results indicated that services provided by GOPC were more often used by female, older, poorer, chronically-ill and less educated population. GOPC participants were also more likely to have visited a specialist or used specialist services (69.7% vs. 52.0%; *p *< 0.001), although this difference in utilization of specialist services disappeared after adjusting for age (55.7% vs. 52.0%, *p *= 0.198). Analyses were also performed to asses the relationship between healthcare settings (GOPCs versus private GPs) and primary care quality. Private GP patients achieved higher overall PCAT scores largely due to better accessibility (Mean: 6.88 vs. 8.41, *p *< 0.001) and person-focused care (Mean: 8.37 vs. 11.69, *p *< 0.001).

**Conclusions:**

Our results showed that patients primarily receiving care from private GPs in Hong Kong reported better primary care experiences than those primarily receiving care from GOPCs. This was largely due to the greater accessibility and better interpersonal relationships offered by the private GPs. As most patients use both GOPCs and private GPs, their overall primary care experiences may not be as different as the findings of this study imply.

## Background

Considerable evidence supports the important role of primary care in the prevention of illness and death, regardless of whether the care is characterized by the supply of primary care physicians, a relationship with a source of primary care, or the receipt of important features of primary care [1]. Good primary care, in contrast to specialist services, is also associated with a more equitable distribution of health within and across populations [2,3]. Overall, both individual and ecological studies have demonstrated that high quality primary care is associated with better health outcomes [4-6].

Early reports of the World Health Organization (WHO) defined primary care as "the first level of contact of individuals, the family and the community with the national health system, bringing health care as close as possible to where people live and work" [7]. The US Institute of Medicine defines the functions of primary care as the "provision of integrated, accessible health care services by clinicians who are accountable for addressing a large majority of personal health care needs, developing a sustained partnership with patients and practicing in the context of family and community" [8]. Defined by service characteristics, primary care represents "care that is ambulatory and directly accessible to patients, with a generalist character, situated in the community that it serves and with a focus on the individual in his or her home situation and social context." [9-11]. In 2008, the World Health Organization urged all countries to strengthen their primary care systems and to use primary care as a model to provide care that is fair and efficient [12,13].

Researchers have operationalized [9-11] the attributes that define primary care and suggest that quality primary care should involve at least the following five attributes: 1) First contact accessible care; 2) Continuity of care - addressing the patient's health needs over time; 3) Care centered on the patient over time - takes into account the personal and social context in the treatment; 4) Comprehensiveness of care - providing care for common problems including providing curative, rehabilitative and supportive care, as well as health promotion and disease prevention; and 5) Coordination of care - seamless care so that when patients are referred elsewhere the advice they receive is integrated into their care. These primary care attributes have been used as measures of the quality of primary care services and studies have shown that the presence of each of these attributes of primary care improves both the effectiveness and efficiency of care [14-17].

In Hong Kong, fees for all services at public hospitals and clinics are heavily subsidized by the government. Public inpatient hospital charges are 100HKD (1USD = 7.8HKD) per day. Over 90% of all in-patient services (in terms of the number of bed days) in Hong Kong are provided by public hospitals [18]. There is a copayment of 100HKD for the first attendance at a specialist outpatient consultation and 60HKD for each subsequent specialist outpatient consultation. There is an additional drug charge of 10HKD per prescription/drug item. For a generalist consultation, there is a copayment of 45HKD with no medication copayments in the public sector general outpatient clinics.

The Food and Health Bureau (FHB) of the Government is responsible for overseeing the health care system. The Department of Health (DH), which reports directly to the FHB, is mainly responsible for performing public health and health promotion functions. It also provides direct care in four specific service areas including maternal and child health services, student health services, social hygiene and dermatological services and elderly health services.

The Hospital Authority (HA), which also reports to the FHB, manages all 44 publicly funded hospitals (including their specialty clinics) and 74 general outpatient clinics (GOPCs) through seven geographic clusters. The role of the GOPCs is to provide access and quality clinical care to needed primary care services for the financially vulnerable, the elderly and patients of chronic diseases [19]. Most GOPCs are located in the community, though a few are located within public hospitals. Public specialist clinics are located within hospitals. Both public GOPCs and hospitals of the Hospital Authority share the same electronic patient record system.

There is no requirement for a doctor to have any training in family medicine to practise general practice or family medicine in Hong Kong. Of the total of 11,950 registered medical doctors in Hong Kong in 2007, only 196 doctors are believed to be members or fellows of the Hong Kong College of Family Physicians, signifying that they have received formal training in family medicine [20].

In the GOPCs, about one-third (30%) of doctors do not have any formal training in family medicine. The remainder are either family medicine trainees or trainers in family medicine (trainees form the larger proportion). Indeed, the Hospital Authority is the key provider of community training in family medicine in Hong Kong.

Physicians in GOPC are salaried, and family medicine trainees or trainers are paid the same salary as their counterparts in other specialties. GOPCs usually operate from 9 am to 5 pm on weekdays and 9 am to 1 pm on Saturday. A small minority of GOPCs also provide general medical services outside the usual opening hours, on evenings (6 pm - 10 pm) and on the mornings of public holidays. GOPCs have a set number of appointment slots per day, a proportion of which are open appointment bookings in each clinic session.

The private sector is the major supplier of primary care, providing about 70% of out-patient consultations [18].

The payment system in the private sector is also based on a fee for a service, though payment charges are set by individual medical practitioners, and are not subject to government regulation or guidance. All private services are either paid for by the patients themselves or by private insurance (individual or employer's insurance) with either submission of claims forms to insurance companies by doctors (among group medical insurance plans purchased by the employers) or payment of fee by the patients first with subsequent reimbursement by the insurance companies (mostly by individual insurance plan). Insurance plays a relatively small role in the overall health care system, and does not cover mental health problems, chronic diseases or preventive services.

In the private sector, there is no control over what kind of doctors can practice as a general practitioner. For example, a new graduate of medicine with one year of internship can work in a private solo practice. Most private practices have only one physician. In contrast, the income of solo private practices or group practices depends on the number of patient visits and the price charged by the practitioner. The opening hours for private facilities are at the discretion of the practitioner; some are open for 24 hours. There are very few physicians (and none in the GOPCs) who make home visits.

In public hospitals, access to specialists is only through referral from primary care doctors from both the public and private sector or from other specialists, although no referral is needed when specialists ask patients to return for follow-up (There is no limitation on the number of visits to specialists).

Visits to private specialists can be made without any referral and patients can directly visit private specialists on their own accord. Preventive care services in Hong Kong such as immunizations for children and influenza vaccination for elderly are provided free of charge at GOPCs as well as in the maternal and child centres of the Department of Health. Pap smear examinations are available at the Department of Health clinics, but are subject to a service fee of a few hundred dollars. There are no territory-wide population screening programs in Hong Kong and there are no patient registers in any type of facility.

Although the services provided by GOPCs and Department of Health clinics may overlap, there is no or little communication between the GOPCs and the services provided by the Department of Health. It is not uncommon for patients to use both services for similar problems (e.g. the elderly can be seen by both DH clinics and GOPCs). This fragmentation in the provision of primary care services means that there is no well established policy or territory wide primary care network to effectively perform the gate keeping or continuity of care functions. Moreover, about half of all specialists work in the private sector and most provide both specialty and general practice care. There is no registry of private practicing primary care physicians and their number and function are unknown.

In Hong Kong, although the mission of GOPCs is to serve the underserved, no studies have evaluated the extent to which they do so. Such efforts to evaluate or better understand how the current GOPCs are able to serve the most vulnerable populations are very timely for Hong Kong. The recent health care reform consultation document released in 2008 [18] and the recently released 2009 policy address both proposed [21] enhancing primary care services through incorporating more preventive care services and providing comprehensive primary services in local communities by coordinating other community based health care and social services for the vulnerable populations and the elderly.

Our study was conducted to compare the current primary care quality of general outpatient clinics with those of other private providers of primary care in Hong Kong using an internationally recognized measure to evaluate primary care performance. Its findings should provide useful information for policy makers and health service researchers in Hong Kong.

## Methods

A stratified random telephone survey was conducted on residents aged 18 or above in Hong Kong by the Centre for Epidemiology and Biostatistics of the Chinese University of Hong Kong. Three major geographic regions were stratified and telephone numbers were randomly selected from a telephone directory. Respondents were selected by a modified "last birthday" method for each contacted household. This was done to minimize over-representation of housewives and the elderly in the sample. The interviews were conducted between 6:00 pm and 10:30 pm to avoid oversampling of the unemployed or homemakers. All interviews were performed by trained interviewers. Calls were attempted three times before the telephone number was classified as invalid. No interviews were attempted in non-Chinese-language households (about 1% in Hong Kong), commercial numbers, or fax numbers.

The individual who answered the phone was told that the study would collect information about the quality of primary health care services in Hong Kong, and that participation would help the government improve primary health care services in Hong Kong. A household member aged 18 or above whose past birthday was closest to the day of the interview was chosen to participate in the study.

We aimed to obtain 1000 completed surveys for the current study. This sample size was calculated based on findings from a previous paper that compared the PCAT scores between an HMO population and a CHC population [22]. We estimated the means and standard deviations for each primary care measure conservatively. The largest sample size required was 300 per group based on the sample size calculation (α = 0.05 and a power of (1-β) = 0.9). Since only 30% of primary care is provided by the public sector in Hong Kong, we have increased our sample size (which drew randomly from the population) to account for unequal private, public distribution of primary care services in Hong Kong. We therefore calculated that around 1000 completed surveys would provide the required power for analysis.

**Figure 1:**
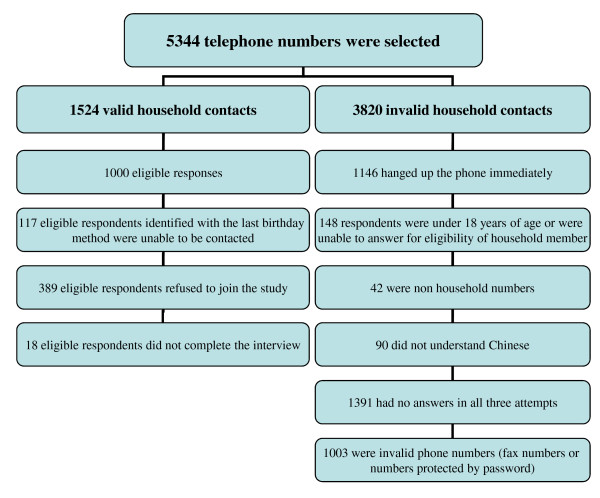
**Details of participant selection**.

The details of participant selection are set out in figure 1. A total of 1524 valid household contacts were made. Of these contacts, 117 respondents with the last birthday identified to could not be contacted after three attempts, 389 respondents refused to join the study, and 18 did not complete the interview. The overall response rate (defined as the number of completed interviews divided by the total number of valid household contacts) was 65.6% (1000/1524). The study was approved by the Survey and Behavioural Research Ethics Committee of the Chinese University of Hong Kong.

### Measurement

Our study used the Primary Care Assessment Tool-Adult Edition (PCAT-AE) for data collection. PCAT-AE was developed by the Johns Hopkins Primary Care Policy Centre to measure patients' experiences with their source of care. The tool focuses on the experience of consumers' primary care experiences rather than their satisfaction with care. The PCAT has been validated [23] and shown to have excellent reliability and validity with respect to the domains of primary care. The English version of the PCAT-AE was translated by a PhD graduate in anthropology and was back translated into Cantonese Chinese. Its content was further reviewed by 2 family medicine academics, 2 family doctors and 1 public health academic and one research associate with experience in conducting telephone survey to check for its face and content validity. It was pilot tested on 20 adults from the general population with further modifications before use for the territory wide telephone survey.

### Domains of primary care

The original questions in the PCAT survey were modified for the version used in the current survey after piloting with local participants. The questions "Is there a doctor that knows you best as a person" and "Is there a doctor that is most responsible for your health care?" were not used as participants were confused and had difficulty in understanding the meaning of these two questions in Hong Kong. As a result, during the actual survey, the participants were only asked whether they had a doctor that they usually consulted first if they were sick or needed advice about their health. It was explained that this did not include "a doctor in the A & E department of a hospital". Eight scales in the PCAT were used: first contact-accessibility, first contact-use (first contact domain), continuity of care (longitudinality), coordination of services (coordination domain), comprehensiveness-services available, comprehensiveness of services received (comprehensiveness domain), community and family centeredness and community orientation (derivative domain). First contact care is defined by accessibility to and use of services for each new problem or each new episode of a problem for which people utilize care. Longitudinality defines the characteristics of the interpersonal relationship between the provider of regular source of care and the patient. Coordination of care includes some form of continuity, either interpersonal or informational (through electronic health records for example) or both, as well as integration of problems addressed elsewhere into the total care of the patients. Comprehensiveness measures the range of all types of health services or the arrangement of all types of health care services within the clinics [6]. In addition, two domains related to the core domains were included in the PCAT: community orientation refers to the providers' involvement of community activities and their understanding and knowledge of the needs of the community and family centeredness refers to the inclusion of family health concerns and problems. These primary care attributes or domains are consistent with both the WHO and Institute of Medicine definition of primary care [7,8].

The Likert scale questionnaire was scored on a 1 to 4 scale, with 1 indicating "definitely not", 2 indicating "probably not", 3 indicating "probably", 4 indicating "definitely", and 2.5 indicating "not sure/cannot remember". The total score for each domain was calculated by summing (with reverse coding whenever appropriate) the values for all items under each domain. The total score for overall primary care achievements was derived by summing all the values from each domain.

In addition to the questions from the PCAT-AE, additional questions were added to collect data on socio-demographic characteristics (household income, insurance coverage, education, geographical districts, age and gender) and other characteristics of primary care provision that are relevant for the provision of primary care in Hong Kong. These included asking if the person had ever visited other doctors beside their primary care practitioners (e.g. TCM practitioners), whether they had self reported diagnosed chronic diseases (from a checklist of 11), their main provider of chronic disease management, and the number of visits from the chronic disease management provider in the prior 12 months. Questions to explore the source and type of preventive care (influenza vaccination for participants over 65 years of age, papanicolaou test for female participants over 18 years of age and doctor administered breast examination for female participants over 40 years of age) were also included in the study; however, only findings obtained from the PCAT-AE are the subject of this paper.

### Statistical analysis

Our analysis was similar to that performed by Shi *et al *[22]. The aim of the analysis was to compare the achievement of primary care quality attributes of the GOPCs with those of the private GPs in Hong Kong. We also compared the socio-demographic information of our participants with those of the Hong Kong population in general. The chi-square test was used to test for differences in demographic and service use characteristics of the two populations (GOPC vs. private GP). Comparisons were made with respect to individual and total primary care attributes between the GOPCs and the private GPs. Differences in the means of primary attribute scores between the GOPCs and the private sector GPs were also compared using the independent samples t-test. Analysis of covariance was conducted for comparison after adjustments were made for socio-demographic and health care characteristics. Multiple linear regression analysis was conducted to examine the association between health care settings, health care characteristics, socio-demographic status and primary care assessment score (PCAT). The data were analyzed using SPSS 16.0.

## Results

The socio-demographic information of our participants was compared with those of the Hong Kong general population. The participants of our telephone survey were older and were more likely to have higher education.

With respect to health service utilization, 201 of the 1000 respondents (20%) reported GOPC, 759 participants (75.9%) reported a private GP and thirty-five (3.5%) reported Traditional Chinese Medicine Practitioners as their main primary care practitioner. Five respondents (0.5%) reported "others". About four in five respondents in both types of services had visited Traditional Chinese Medicine practitioners at least once. Many participants had visited other doctors than the one they identified as their primary care practitioner. Almost all the respondents (96%) who identified GOPC had also visited private GPs, and more than 40% had done so in the past 12 months alone. Over 4 in 5 (82.6%) of the respondents who identified a private GP as their primary care practitioners had visited a GOPC for medical services, and 23% had visited a GOPC doctor within the past 12 months.

About a quarter of respondents reported doctor diagnosed chronic conditions, of which 58% reported hypertension, 22.7% diabetes, 9.8% heart disease and the rest other diseases. Among those with chronic conditions, 23.5% identified GOPC as their main provider for chronic disease management; 13.6% identified a private GP and about half identified a government-operated specialist outpatient clinic as their main providers of chronic disease management. That is, those with reported chronic diseases were much less likely to receive their chronic care management from their reported private primary care provider.

Table 1 shows the socio-demographic and health care service characteristics among patients in GOPCs and private GP settings. In general, GOPC participants were more likely to be female (58.2% vs. 51.0%; *p *= 0.04), more likely to be over age 60 (45.8% vs. 12.3%; *p *< 0.001), more likely to have secondary or below education (87.1% vs. 61.6%; *p *< 0.001), more likely to have lower income (<HK $20,000 household income) (76.5% vs. 45.0%; *p *= 0.001), and more likely to report having a chronic condition (49.3% vs. 19.2%; *p *< 0.001). GOPC participants were more likely to have visited their doctors than private GP participants. They were also more likely to have visited a specialist or used specialist services (69.7% vs. 52.0%; *p *< 0.001), although this difference in utilization of specialist services disappeared after adjusting for age (55.7% vs. 52.0%, *p *= 0.198).

**Table 1:** Comparison of socio-demographic characteristics and healthcare services use among adult patients in different healthcare settings

	GOPC(%)	GP(%)	*p *value
	(n = 201)	(n = 759)	
*Sociodemographic characteristics*			
Gender			0.04
Male	84 (41.8%)	372 (49.0%)	
Female	117 (58.2%)	387 (51.0%)	
Age			<0.001
<60	109 (54.2%)	666 (87.7%)	
≥60	92 (45.8%)	93 (12.3%)	
Education			<0.001
Secondary and below	175 (87.1%)	465 (61.6%)	
Above secondary	26 (12.9%)	290 (38.4%)	
Income			0.001
Low income	130 (76.5%)	297 (45.0%)	
High income	40 (23.5%)	363 (55.0%)	
Insurance			<0.001
Yes	38 (18.9%)	414 (54.8%)	
No	163 (81.1%)	341 (45.2%)	
			
*Access to health care*			
How often do you go to your doctor's			0.01
0 time	28 (13.9%)	125 (16.5%)	
1-2 times	78 (38.8%)	324 (42.7%)	
3-4 times	42 (20.9%)	193 (25.4%)	
5-6 times	18 (9.0%)	57 (7.5%)	
More than 7 times	35 (17.4%)	60 (7.9%)	
Have you ever visited specialist or used special services?			<0.001
Yes	140 (69.7%)	395 (52.0%)	
No	61 (30.3%)	364 (48.0%)	
Adjusted special services frequency*			
Have you ever visited specialists or used special services?			0.198
Yes	55.7%	52.0%	
No	44.3%	48.0%	
Have you ever been diagnosed any chronic diseases by a western doctor?			<0.001
Yes	99 (49.3%)	146 (19.2%)	
No	102 (50.7%)	613 (80.8%)	
Adjusted chronic disease frequency*			
Have you ever been diagnosed any chronic diseases by a western doctor?			0.005
Yes	27.9%	19.0%	
No	72.1%	81.0%	

*Adjusted by age using private GP patients' age structure as reference

Table 2 presents the results of the comparison of PCAT scores among participants visiting GOPCs and private GPs. Unadjusted domain scores indicated that GOPC patients reported poorer experiences in all domains except for coordination of care. The results of a Mann Whitney test, which was performed as a sensitivity analysis, were also consistent with the t-test's results. Adjustment for income, insurance, education level, age, gender, use of specialist service and the presence of chronic medical conditions attenuated the differences although those using private physicians still scored significantly better for first contact care, continuity of care and the total score (Table 2).

**Table 2:** Comparison of adjusted primary care assessment scores among adult patients in different healthcare settings

Primary care domains	Range of values	GOPC Score Mean(SE)	GP Score Mean(SE)	*p *value
Adjusted primary care achievement*				
First Contact (Utilization)	(3-12)	8.87 (0.15)	9.21 (0.07)	0.048
First Contact (Accessibility)	(4-16)	6.88 (0.15)	8.41 (0.07)	<0.001
Continuity of Care	(4-16)	8.37 (0.18)	11.69 (0.08)	<0.001
Coordination of services	(4-16)	9.47 (0.31)	9.67 (0.17)	0.584
Coordination (Information System)	(4-12)	8.00 (0.10)	8.06 (0.05)	0.660
Comprehensiveness: Service available	(6-24)	14.97 (0.31)	15.71 (0.15)	0.046
Comprehensiveness: Service provided	(6-22)	12.14 (0.26)	12.05 (0.13)	0.764
Family Centeredness	(3-12)	7.74 (0.15)	8.07 (0.07)	0.056
Community Orientation	(3-10.5)	4.79 (0.11)	4.71 (0.05)	0.541
Primary care total score	(50.5-133)	77.03 (0.80)	83.26 (0.38)	<0.001

*Primary care scores adjusted for gender, age, education level, income, insurance, use of specialist services, chronic disease conditions.

Results from the linear regression analysis (Table 3) indicated that patients who described a private GP as their main primary care provider had higher overall PCAT scores (*p *< 0.001). Among health care use measures, factors positively associated with primary care quality included having visited any kind of specialists or specialist services (*p *< 0.001), having been diagnosed with a chronic condition by a western doctor (*p *= 0.027) and having private medical insurance (*p *= 0.046), Among the socio-demographic characteristics, having above secondary education and having a higher income (HKD $20,000 or above) was positively associated with primary care quality as measured by the PCAT score (*p *= 0.006 and *p *= 0.001 respectively). Similar results were obtained when ordinal logistic regression was used as a sensitivity analysis, except that gender was a statistically significant predictor in the ordinal logistic regression model but not in the linear regression model. According to the ordinal logistic regression results, male patients achieved higher overall PCAT scores (*p *= 0.018).

**Table 3:** Linear regression analysis between primary care assessment score and healthcare setting/socio-demographic characteristics

Dependent variable: Primary care achievement (total score)	B	SE	*p *value
Intercept	67.598	1.091	<0.001
Health care settings			
GOPC	-		
Private GP	5.997	0.918	<0.001
Health care service use			
Visited specialist			
No	-		
Yes	11.203	0.734	<0.001
Chronic disease condition			
No	-		
Yes	1.762	0.909	0.027
Medical insurance			
No	-		
Yes	1.319	0.782	0.046
Socio-demographic characteristics			
Gender			
Female	-		
Male	0.898	0.681	0.094
Age			
Less than 60 years old	-		
60 years or older	0.475	1.098	0.333
District			
New Territory	-		
Hong Kong Island	0.800	0.879	0.182
Kowloon	-0.844	0.786	0.142
Education			
Secondary or below	-		
Above secondary	1.994	0.788	0.006
Income			
Below 20K	-		
20K or above	2.348	0.797	0.001
Model Statistics			
R Square	31.7%		
Adjusted R Square	30.8%		
F value	37.745		

An additional analysis was conducted to compare patients who have only visited General Outpatient Clinics (GOPCs) with those who have only visited private GPs in the past year. This analysis was conducted because a large proportion of patients visited both types of service provider. It was found that 111 people only visited GOPCs in the past year, and 483 people only visited private GPs. According to our results, people who only visited private GPs had significantly better primary care experiences than those who only went to GOPCs in "First Contact (Accessibility)" domain (*p *< 0.001), "Continuity of Care" domain (*p *< 0.001), "Centeredness" domain (*p *= 0.014), "Community Orientation" (*p *= 0.042), and the total score (*p *< 0.001) which are similar to results obtained from our original analysis.

## Discussion

This study provides a useful foundation for understanding the complex primary care system in Hong Kong. Our results showed that patients of private GPs in Hong Kong reported receiving better primary care experiences than those who reported receiving their care at GOPCs, largely because of the greater accessibility and better interpersonal relationships of GPs. These findings should be interpreted carefully, however, as the telephone survey may have introduced bias by reflecting the views of people more likely to respond to this kind of survey. We have also compared the characteristics of our respondents to those of data obtained from the Hong Kong Census, and concluded that our respondents are similar to the Hong Kong population in general except for the likelihood of receiving additional education.

International experience [24] from Europe (although not the US) consistently shows that general practice care is usually pro-poor. In other words, findings from these countries show that the poor tend to use general practice care more often than specialist care, and there is evidence showing that GP care can reduce social inequity resulting in better distribution of health [4]. On the other hand, studies in Hong Kong indicated the opposite. Lu *et al *[25] showed that after controlling for a given need, there is a bias for general practitioner services to be used more often by the better off in Hong Kong. In other words, there appears to be unequal treatment for the same need, as better-off patients in Hong Kong are more likely to identify with private general practitioners.

Our findings suggest that the services provided by private GPs, which are used more often for the younger, richer, less chronically-ill, and more educated population may, at the least, be more accessible as reflected by the PCAT domain scores and with better interpersonal relationships. However, the issue of joint usage of both private and public general practice services by people in Hong Kong makes it difficult to conclude that this is indeed the case, although our sensitivity analysis gave further support for our findings. Further research on the reasons for the high level of use of both types of services is warranted.

People diagnosed with a chronic condition or who had visited specialists were found to have higher primary care scores than those who did not have chronic disease or had not visited a specialist, regardless of whether they identified with private or public facilities. Further sub-analyses showed that better coordination in terms of better information systems may account for the higher PCAT scores among those who had visited specialists or with a chronic condition, at least in the public sector. This is consistent with our previous experience as patients with chronic conditions in the public sector are given a pocket size handheld booklet that records their medications, chronic conditions and investigation results. As a result, patients can bring this handheld record and show it to any health care providers when needed. In addition, there is a common electronic health record system across all general outpatient clinics and the hospital authority hospital system.

Our findings also showed that those who considered GOPCs as their primary health care practitioners were more likely to have visited a specialist or used specialist services, although the relationship disappeared after adjustment for the presence of chronic diseases. This implies that the greater number of specialist visits by GOPC patients was mainly due to the fact that there was a larger proportion of patients with chronic diseases in the GOPC populations. In this study, the result showed that participants with higher PCAT scores (who identified either GOPC doctors or private GPs as their primary care providers) were more likely to have visited specialists (after adjustment for the presence of chronic diseases and type of primary care practice). This is also likely to be related to the higher information system domain scores reported by those who have used specialist services, consistent with the existence of a common electronic patient record system. Furthermore, private providers who provide care for patients who also consult public doctors (whether GOPCs or hospitals) can access patients' public electronic health records with their consent. Since most specialist care in Hong Kong is provided by the public sector and the public sector is equipped with a well developed, central electronic patient record system, the finding that "having visited specialists" was associated with higher PCAT scores was not surprising.

Our data indicated that although a small proportion (3.5%) of respondents identified Traditional Chinese Medicine (TCM) practitioners as their primary care providers, 33-40% of participants who considered western doctors as their major primary care practitioners had also visited TCM practitioners in the past 12 months. This finding is consistent with our previous studies which showed that TCM is the major form of complementary and alternative medicine in Hong Kong [26]. Middle aged chronic disease patients are more likely to use western and TCM concurrently [27], and the rationale for making such choice could be the desire to experience a stronger interpersonal relationship with TCM practitioners [28], to reduce the side effects of western medications, or to revitalize the body by using TCM tonics [Chung, Lau, Mok, Yeoh and Griffiths: View on traditional Chiense medicine amongst Chinese population: A systematic review of qualitative and quantitative studies, submitted]. The effects of consulting both Western and TCM practitioners in the primary care settings on the continuity and coordination of care warrants further investigation [29].

Our study supports the need to improve primary care in Hong Kong by increasing community GP accessibility and to further develop the primary care role of GOPCs. The information should be useful for the government's healthcare reform policy. Initiatives to provide multidisciplinary team support and the development of electronic linked records and develop the roles of nurses are all under consideration. It is apparent that GOPCs in Hong Kong do not play the same role as, for example, CHCs in the US [17], in that they do not provide uniformly better care than private practices. Several possible reasons may account for the differences in primary care experiences in CHCs in the US compared with GOPCs in Hong Kong. Evidence shows that the level of enabling services such as transport, translation, child care available in a CHC (US) may help improve health outcomes such as infant mortality including those for pregnant women [30], and better coordination with other community wide multi-sectoral initiatives that are associated with health promotion such as the Healthy Start, which have been linked to better health outcomes [31,32]. Social services are not integrated with the GOPCs in Hong Kong and there are competing primary care services from other government departments (Department of Health) that may affect both continuity and coordination of care. The role of community nurses and paucity of primary care team development are further barriers.

Several limitations were identified in our current study. First, as discussed, our methods of using telephone survey may have limited the representativeness of our findings. Furthermore, since reports of primary care experiences in the private sector may reflect responses of doctors to patients' demands rather than needs, the higher continuity of care that was seen in the users of private doctors may reflect only the fact that private doctors were more responsive to patient centred demand on services. However, this study did not evaluate whether these services were cost effective and evidence based to. This is a subject requiring further research. Doctor shopping is common in Hong Kong [33]. As a result, participants may have found it difficult to identify their usual source of primary care. Secondly, data from this study were cross-sectional and did not allow for the demonstration of causality. Thirdly, we were unable to adjust for "clustering" effects with respect to primary care practice provision as we did not collect such data. A standard multiple linear regression model was fitted to our data, instead of an ideal multi-level model of patient "within" primary care provided. Fourthly although we have adjusted for several variables, unknown characteristics could have mediated the relationship between type of setting and the PCAT scores. There also may be fundamental differences between the GOPC and private GP patients in attitudes toward health care practices. Fifthly, we did not control for the possible effect of different frequency of use in the two groups of respondents. People who are particularly high users of services are over-represented in surveys based on patient visits. Therefore, all of our findings should be interpreted as experiences of people making visits, not people in general. Finally, we have only used the first question (A1) in the PCAT to define the primary care provider and this may have affected our results and made it difficult to compare our results with those of previous studies that used all three questions.

## Conclusions

In summary, we have shown that respondents who identified GOPCs as their regular source of primary health care provision had poorer scores for primary health care attributes, largely due to limited accessibility and patient-focused care over time. As the government in Hong Kong is currently considering improving primary care service provision, attention should be paid to the role and function of GOPC services. The relative role of primary care providers and specialists, especially in the care of people with chronic disease, requires additional exploration. Further research will be needed to determine whether forthcoming reforms succeed in improving primary health care services, especially for the socially and medically vulnerable.

## Competing interests

The authors declare that they have no competing interests.

## Authors' contributions

Please see sample text in the instructions for authors. SYSW, KK, SMG and TC designed the study. SYSW, KK and MCSW participated in data acquisition. SB, SYSW, SVL, KK, WG and VCHC are responsible for data analysis and interpretation. SYSW and MCSW drafted the article. SB, KK, SMG, VCHC, WG and SYSW revised the draft for intellectual content. All authors helped in final approval of the completed article. SB was also responsible for setting the international scene and making comparisons, particularly with the US in this paper.

## Pre-publication history

The pre-publication history for this paper can be accessed here:

http://www.biomedcentral.com/1471-2458/10/397/prepub
